# Specific Contributions of Basic Numerical Skills and Working Memory to Mathematics Skills in Primary School

**DOI:** 10.3390/jintelligence14070115

**Published:** 2026-06-24

**Authors:** Vroni Hischa, Frank Niklas, Korbinian Moeller

**Affiliations:** 1 Chair of Developmental and Educational Psychology, Catholic University of Eichstaett-Ingolstadt, 85072 Eichstaett, Germany; 2 Department of Psychology, LMU Munich, 80802 Munich, Germany; niklas@psy.lmu.de; 3 Institute of Psychology, UMIT TIROL, Private University for Health Science and Technology, 6060 Hall in Tyrol, Austria; korbinian.moeller@umit-tirol.at; 4 Department of Mathematics Education, Loughborough University, Loughborough LE11 3TU, UK; 5 LEAD Graduate School & Research Network, University of Tuebingen, 72074 Tuebingen, Germany

**Keywords:** mathematics skills, primary school, non-symbolic numerical skills, symbolic numerical skills, working memory, developmental changes

## Abstract

The development of mathematics skills is driven by domain-specific and domain-general cognitive predictors, like non-symbolic and symbolic numerical skills and working memory (WM), respectively. However, differential and potentially changing contributions of these predictors across primary school years have hardly been investigated jointly. Therefore, we assessed 486 German primary school children from first to fourth grade (i.e., 6 to 11 years old) on (non-)symbolic magnitude comparison, approximate addition, number line estimation, WM, nonverbal reasoning, and their mathematics skills in a cross-sectional study. Results of a multi-group path model indicated that number line estimation and WM were significant predictors of mathematics skills in all grade levels, and nonverbal reasoning was a significant predictor of mathematics skills in first, third, and fourth grade. Pairwise comparison of path coefficients further revealed that contributions of number line estimation were significantly stronger in third grade than in first and second grade. In sum, these results suggest that complex symbolic numerical skills and WM contribute to the development of mathematics skills in primary school, potentially reflecting effects of formal mathematics instruction.

## 1. Introduction

The development of mathematics skills is driven by domain-specific numerical and domain-general cognitive predictors (see Passolunghi & Lanfranchi, 2012; Martin et al., 2014). The processing of both non-symbolic and symbolic magnitudes is regarded as a domain-specific numerical predictor of mathematics skills in different theories of mathematics development (e.g., Siegler & Braithwaite, 2017; von Aster & Shalev, 2007). Typically assessed by magnitude comparison, approximate arithmetic or number line estimation tasks, the processing of non-symbolic and symbolic magnitudes was repeatedly observed to significantly predict mathematics skills in general (e.g., M. Schneider et al., 2017; M. Schneider et al., 2018; Chen & Li, 2014). In addition, working memory (WM) was consistently identified as a significant domain-general predictor of mathematics skills in a growing number of studies (e.g., Raghubar et al., 2010; De Smedt et al., 2009).

Even though there is accumulating evidence on the relevance of these variables for mathematics skills (see, e.g., De Smedt, 2022 for a review of meta-analyses), studies jointly evaluating differential and potentially changing influences of these variables on mathematics skills across primary school years are still scarce. However, this seems particularly important when considering the model of mathematics development by von Aster and Shalev (2007). This model proposes contributions of domain-specific numerical skills such as non-symbolic and symbolic numerical skills to the development of mathematics skills more generally. As a first step, the model assumes that the innate ANS enables an initial understanding of quantity, which serves as a necessary foundation for associating quantities with corresponding symbolic representations. As a second step, the model posits that children learn the verbal number system and thus number words as a first form of symbolization. In a third step, children master the Arabic number system as a second form of symbolization. These two forms of symbolization are, in turn, essential for developing a mental number line in the fourth step of the model. Furthermore, according to von Aster and Shalev (2007), the described development of domain-specific numerical skills is accompanied by an underlying development of domain-general abilities, like WM and general cognitive abilities. For instance, increasing WM capacity enables better information processing and therefore supports the development of the mental number line representation.

In sum, the model by von Aster and Shalev (2007) posits that it is essential to consider domain-specific numerical predictors such as non-symbolic and symbolic numerical skills as well as domain-general predictors like WM jointly because of their close association during the development of mathematics skills. Ideally, this is done across different grade levels to capture their contributions as well as potential changes in these across development.

Accordingly, the aim of the current study was to evaluate contributions of non-symbolic (assessed using non-symbolic magnitude comparison and approximate addition) and symbolic numerical skills (assessed by symbolic magnitude comparison and number line estimation) as well as WM to the development of mathematics skills during primary school years.

### 1.1. Contributions of Non-Symbolic Numerical Skills

Processing non-symbolic numerical information is typically associated with the so-called approximate number system (ANS). The ANS is assumed to reflect an innate, nonverbal system that enables imprecise (approximate) representations of non-symbolic quantities. This system enables the comparison of non-symbolic quantities (e.g., dot patterns, see Halberda & Odic, 2015; Starr & Brannon, 2015). The ANS is seen as a building block for the development of more complex mathematics skills in primary school children (Feigenson et al., 2013; M. Schneider et al., 2017; Chen & Li, 2014; Malone et al., 2020; Mazzocco et al., 2011). In general, a more accurate ANS is assumed to reflect more accurate representations of non-symbolic quantities, which may support further development in mathematics, especially at an early stage of the development of mathematics skills (Chen & Li, 2014; Feigenson et al., 2013). The two most common tasks to assess the ANS are non-symbolic magnitude comparison and approximate addition.

In non-symbolic magnitude comparison tasks, typically, two arrays of dots have to be compared according to the quantities they reflect without counting (e.g., Halberda et al., 2008). In approximate addition tasks (e.g., Barth et al., 2005), two dot arrays are presented successively and disappear after a short period of time while participants have to add the respective quantities reflected by these dot arrays. Typically, another dot array is then presented as a probe and participants have to indicate whether the sum of the two previously presented dot arrays is smaller or larger than the presented probe.

Contributions of the ANS to the development of mathematics skills are discussed controversially as previous findings are not consistent (De Smedt et al., 2013; Chen & Li, 2014). This may be explained by methodological differences, such as task characteristics and the control of domain-general cognitive predictors like general cognitive abilities and/or WM (De Smedt et al., 2013; Dietrich et al., 2015). In particular, different ANS tasks pose additional demands on domain-general cognitive abilities to different extents (Dietrich et al., 2015). For example, when the dot arrays are presented successively instead of simultaneously, this increases demands on WM. Accordingly, Dietrich et al. (2015) pointed out that, especially when interested in the relationship between ANS and mathematics skills, the control of domain-general cognitive abilities such as general cognitive ability and WM is important as these considerably correlate with mathematics skills themselves. However, findings on whether non-symbolic numerical skills predict mathematics skills in primary school children remain heterogeneous even when controlled for such domain-general abilities (Coolen et al., 2022; Gimbert et al., 2019; Malone et al., 2020, 2019).

Importantly, accuracy of the ANS increases with age (Halberda & Feigenson, 2008; Halberda et al., 2012). This means that, for instance, children at the age of three years are able to correctly distinguish arrays of objects with a ratio of 1.33 (e.g., 12 vs. 16 dots) whereas adults are able to distinguish arrays with a ratio of 1.1 (e.g., 10 vs. 11 dots) (Halberda & Feigenson, 2008). Therefore, it seems plausible to assume that the role of the ANS may change over the course of development in mathematics. For instance, some previous studies reported that the predictive power of the ANS for mathematics skills decreases from preschool to primary school (Cai et al., 2018; Gimbert et al., 2019). In contrast, others still observed significant associations between ANS and mathematics skills in primary school and even older children (Malone et al., 2020; Fazio et al., 2014; Halberda et al., 2008).

### 1.2. Contributions of Symbolic Numerical Skills

In contrast to non-symbolic numerical skills, symbolic Arabic digits as representations of numerical magnitudes have to be acquired through learning. Nevertheless, similar to the ANS, more accurate symbolic representation of numerical magnitude was found predictive of mathematics performance (e.g., M. Schneider et al., 2017, for a review and meta-analysis). Two tasks typically used to assess symbolic numerical skills are symbolic magnitude comparison and number line estimation.

In symbolic magnitude comparison tasks, typically, two symbolic numbers are presented next to each other and participants then have to compare the presented numbers according to their numerical magnitude (e.g., Obersteiner et al., 2013). Performance in such symbolic magnitude comparison was found to predict mathematics skills (Toll et al., 2015). For instance, Li et al. (2018) found that at age 5, 6 and 7 years symbolic skills significantly predicted mathematics skills, whereas non-symbolic skills only predicted mathematics skills indirectly via symbolic skills at the age of 5 and 6 years. The fact that symbolic numerical skills may be more important than non-symbolic numerical skills is substantiated by recent meta-analyses (De Smedt et al., 2013; M. Schneider et al., 2017). Consequently, once children master the processing of symbolic numbers, contributions of non-symbolic numerical skills for the development of mathematics skills might diminish.

In number line estimation tasks, participants have to locate a target number (e.g., 57) on an unscaled number line with only the start and end point given, for instance, 0 and 100 (e.g., Siegler & Opfer, 2003). Generally, more accurate number line estimation performance was found to be associated with better mathematics skills (M. Schneider et al., 2018). In primary school children, number line estimation performance was repeatedly observed as a significant predictor of concurrent (e.g., Link et al., 2014; Gimbert et al., 2019), but also future mathematics skills (e.g., Geary, 2011; Bailey et al., 2014). Importantly, M. Schneider et al. (2018) observed in their meta-analysis that the association between number line estimation and mathematics skills gets stronger between 4 and 14 years of age.

Similar to suggestions for ANS tasks by Dietrich et al. (2015), the results by Ünal et al. (2024) suggest controlling for general cognitive abilities and WM when examining associations between number line estimation and mathematics skills because number line estimation is a complex task placing higher demands on these constructs (e.g., Link et al., 2014). Considering their influences should prevent overestimating contributions of number line estimation to mathematics skills.

### 1.3. Working Memory

Generally, WM is a cognitive system for short-term storage and manipulation of information (Baddeley, 2003). One of the most prominent models of WM by Baddeley (2003) suggests three components, the central executive (CE) as control and manipulation component and two subcomponents, the phonological loop (PL) for storage of verbal information and the visuo-spatial sketchpad (VSSP) for storage of visual information.

Zheng et al. (2011) investigated the contribution of WM to mathematics skills in second, third, and fourth graders (with the age of 7, 8, and 9 years, respectively). The authors observed significant contributions of WM to solving word problems in mathematics. Moreover, in a longitudinal study, De Smedt et al. (2009) assessed WM of children at the beginning of first grade (with 6 years of age) and their mathematics skills in the middle of first and the beginning of second grade. Again, WM significantly contributed to children’s mathematics skills, both in first and second grade (see also W. Schneider & Niklas, 2017). In particular, CE was a significant predictor of mathematics skills in first and second grade, VSSP was a significant predictor in first grade and PL in second grade. Therefore, these results substantiate the important role of WM for mathematics skills. Taken together, these results suggest that WM in general is important for (the development of) mathematics skills during primary school years. Accordingly, better WM skills seem to facilitate the development of mathematics skills in general (Hornung et al., 2014; Raghubar et al., 2010; Vukovic et al., 2014), contributing as a domain-general predictor of mathematics skills in primary school children (De Smedt et al., 2009; Meyer et al., 2010; Passolunghi et al., 2007; Zheng et al., 2011).

### 1.4. Joint Contributions to the Development of Mathematics Skills Throughout Primary School

From the above, it becomes evident that non-symbolic and symbolic numerical skills as well as WM contribute significantly to the development of mathematics skills in primary school (Feigenson et al., 2013; M. Schneider et al., 2017; M. Schneider et al., 2018; Friso-van den Bos et al., 2013). However, in most previous studies, contributions of these variables were not considered jointly in a way that allows us to evaluate differential and potentially changing contributions of these variables to development in mathematics (see, e.g., De Smedt, 2022 for a review of meta-analyses).

The few studies that do so report heterogenous results (Cai et al., 2018; Finke et al., 2020; Gimbert et al., 2019; Li et al., 2018). For example, Cai et al. (2018) tested children from kindergarten, second grade, and fourth grade (5, 7 and 9 years of age). They found that number line estimation as a symbolic numerical skill and WM significantly predicted different mathematics skills in these age groups. For example, in kindergarten number line estimation predicted early mathematics skills (counting, symbolic number knowledge, and arithmetic), whereas in second and fourth grade number line estimation predicted problem solving in mathematics. WM was a predictor of problem solving in mathematics and early mathematics skills (counting, symbolic number knowledge, and arithmetic) in kindergarten and of problem solving in mathematics and calculation fluency in second grade, but not in fourth grade. These results seem to suggest that WM plays a role at the beginning of formal instruction but becomes less important with ongoing instruction.

However, this is in contrast to results by Gimbert et al. (2019) who investigated kindergarten children and children in second grade (i.e., 5 and 7 years of age, respectively). They substantiated that non-symbolic magnitude comparison predicted mathematics skills only in kindergarten children, whereas number line estimation was a significant predictor in both kindergarten and second grade. Additionally, WM predicted mathematics skills only in second grade children. These results indicate that in kindergarten, non-symbolic numerical skills seem important for mathematics skills, whereas in primary school, WM appears to play a more important role. In contrast, symbolic numerical skills are important in both kindergarten and primary school.

In a recent study, Hischa et al. (2025) compared contributions of non-symbolic magnitude comparison, number line estimation and WM to mathematics skills in preschool and third grade children (6 and 9 years of age, respectively). All of these variables were found to significantly predict mathematics skills in both age groups. Nevertheless, number line estimation was a significantly stronger predictor of mathematics skills in third grade than in preschool. Therefore, these results suggest that number line estimation as a specific symbolic numerical skill is more important in primary school children compared to preschool children.

Taken together, these examples illustrate the heterogeneity of results concerning the contributions of non-symbolic and symbolic numerical skills and WM to mathematics skills. However, the different contributions of non-symbolic and symbolic numerical skills and WM to mathematics skills in different grade levels of primary school are not yet understood properly.

### 1.5. The Current Study

Therefore, the current study aimed at a more comprehensive evaluation of differential contributions of non-symbolic and symbolic numerical skills as well as WM to mathematics skills in primary school to better understand potential developmental changes. Based on the findings described above and the model by von Aster and Shalev (2007), we hypothesized that with schooling (and thus training in symbolic numerical skills), contributions of non-symbolic numerical skills to mathematics skills decrease with increasing grade level (H1). Given that the majority of mathematics instruction in primary school is dealing with symbolic numerical representations, symbolic comparison and number line estimation should be very sensitive to schooling. Thus, we expected symbolic skills and therefore symbolic comparison and number line estimation to become more important predictors of mathematics skills for higher grade levels (H2) (M. Schneider et al., 2018; von Aster & Shalev, 2007; Hischa et al., 2025). Furthermore, based on these assumptions and the results of the review and meta-analysis by De Smedt et al. (2013) and M. Schneider et al. (2017) and the large-scale longitudinal assessment by Braeuning et al. (2021), we further hypothesized symbolic numerical skills to make stronger contributions to mathematics skills than non-symbolic numerical skills in our primary school sample (H3). Furthermore, based on studies reported above and the meta-analysis and review by Peng et al. (2016) and Raghubar et al. (2010), we hypothesized WM to be a robust predictor of mathematics skills throughout primary school (H4). Higher WM capacity in general facilitates information processing and learning. Therefore, and in line with the model by von Aster and Shalev (2007), we expect this contribution of WM also within the development of mathematics skills over primary school years.

## 2. Materials and Methods

### 2.1. Participants

In total, 149 first graders (80 females, *M* = 7 years; 1 month, *SD* = 4 months, range: 6; 5–8; 6 years; months), 110 second graders (50 females, *M* = 8; 2, *SD* = 4 months, range: 7; 4–9; 2), 137 third graders (76 females, *M* = 9; 2, *SD* = 4 months, range: 8; 5–10; 4) and 90 fourth graders (50 females, *M* = 10; 1, *SD* = 6 months, range: 8; 2–11; 11) from eight primary schools in the south of Germany took part in this study. Primary schools and parents were informed about the study via information letters and only children who returned written informed consent and provided assent at testing times took part. The study was approved by the ethics committee of the Catholic University of Eichstaett-Ingolstadt (no. 131-2023).

### 2.2. Materials

#### 2.2.1. Mathematics Skills

To assess mathematics skills, tests of the DEMAT series were used as these are standardized tests based on the mathematics curricula in Germany. Due to time and organizational constraints, it was not possible to administer the DEMAT tests with all subtests (see Appendix A Table A1). Consequently, we decided to consider only those subtests which were completed by at least 89% of the respective subsamples. These were eight subtests of the DEMAT 1+ (Krajewski et al., 2021) for first and second graders (i.e., *Quantities – Numbers, Number Range, Addition, Subtraction, Number Decomposition – Number Composition, Part-Whole Relations, Calculation Chains, and Inequations*), eight subtests of the DEMAT 2+ (Krajewski et al., 2020) for third graders (*Number Properties, Comparing Lengths, Addition, Subtraction, Duplication, Division, Bisection, Calculating with Money*), and six subtests of the DEMAT 4 (Gölitz et al., 2006) for fourth graders (*Number Lines, Additions, Subtractions, Multiplications, Divisions, Comparing Quantities*).

For each participant, the reached percentage of the maximum achievable score across the subtests in the respective DEMAT test listed above was computed and considered as a dependent variable for data analyses. Internal consistency (i.e., Cronbach’s alpha) for the subtests of the DEMAT 1+ fell between α = 0.32 and α = 0.87 for first grade and between α = 0.47 and α = 0.87 for second grade, and for the whole test it was α = 0.89 for first grade and α = 0.83 for second grade, which is comparable to the coefficients reported in the manual by Krajewski et al. (2021). Internal consistency for the subtests of the DEMAT 2+ fell between α = 0.49 and α = 0.84, and for the whole test it was α = 0.92 for third grade, which also is comparable to the coefficients reported in the manual (Krajewski et al., 2020). Finally, internal consistency over all subtests of the DEMAT 4 was α = 0.80 for fourth grade and also comparable to that reported in the manual (Gölitz et al., 2006). All DEMAT tests are speeded and subtests often only contain between two and four items. This may partly explain why Cronbach’s alpha is quite low for some subtests (Krajewski et al., 2021; Krajewski et al., 2020; Gölitz et al., 2006).

#### 2.2.2. Tasks Assessing Non-Symbolic Numerical Skills

The *non-symbolic comparison* task used Panamath (Halberda et al., 2008) on a 14″ laptop. Two arrays of dots were presented for 2000 ms next to each other on each trial. The left array consisted of yellow dots, the right array consisted of blue dots. Dot arrays were followed by a pixel mask for 200 ms. As soon as they knew the answer, children had to press the “F” or “J” key on a QWERTZ-keyboard, which were covered with a yellow sticker or a blue sticker, respectively, to indicate which of the dot arrays contained more dots. After an answer was given, there was an interstimulus interval of 1000 ms before the next trial started. In each array, there were between 10 and 30 dots, preventing the number of dots to be counted within presentation time.

Dot diameter was 36 pixels on average with a maximum variation of 25%. The yellow dot array contained more dots on half of the trials. On the other half of the trials, the blue dot array contained more dots. There were eight trials for each of the five used ratios (3.0, 2.0, 1.5, 1.3, and 1.2) (see Elliott et al., 2019; Libertus et al., 2013a; Libertus et al., 2013b; Libertus et al., 2011; Van Herwegen et al., 2017); this means 40 critical trials in total. Presentation order of individual items was the same for all participants.

At the beginning, oral instructions were given by the examiner, accompanied by three practice trials (ratio 3.0). For these practice trials, participants got feedback from the examiner as to the correctness of their response. When their response was incorrect, the examiner explained why. After that, Panamath started with four additional practice trials with a ratio of 3.0 followed by the 40 critical trials for which no more feedback was given.

On half of the trials, we controlled for area occupied by the dots within the arrays to make sure that the task indeed measured ANS accuracy and not only processing of visual features (Dietrich et al., 2015). In size-controlled trials, the area occupied by the dots in both arrays was equal and average dot size was smaller in the array with more dots than in the array with fewer dots. In trials without size control, average dot size was the same for all dots and thus the area occupied by the dots was proportional to the number of dots. Across all grades, Weber Fraction w and the percentage of correct answers were highly correlated (all r ≥ −0.93, all p < .001). Hence, we focused on the percentage of correct answers in all further analyses as a measure for *non-symbolic comparison*. Internal consistency was α = 0.85 for first grade, α = 0.48 for second grade, α = 0.58 for third grade, and α = 0.25 for fourth grade. Low reliabilities for tasks assessing ANS in children are common (e.g., Dietrich et al., 2015; Krajcsi et al., 2024). Therefore, our results concerning non-symbolic comparison should be interpreted with caution.

**Figure A1:**
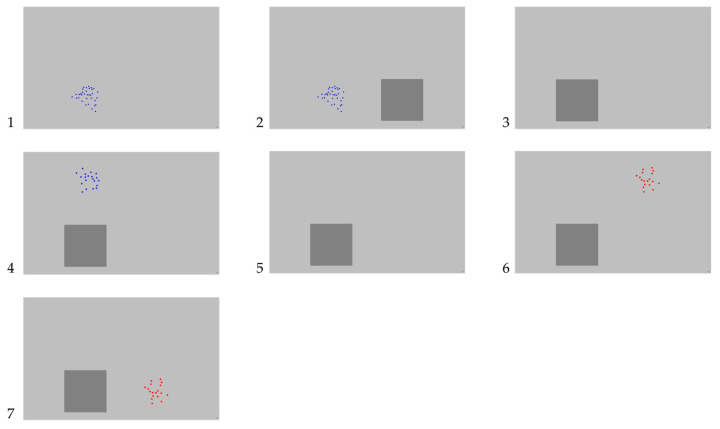
*Approximate addition* task based on Barth et al. (2005). *Notes*. Pictures are numbered in the order of presentation.

The second non-symbolic task was an *approximate addition* task presented via PowerPoint (see Appendix A Figure A1), similar to a task used by Barth et al. (2005). Answers had to be given orally and were recorded by the examiner. On the screen of a 14″ laptop, a blue dot array was shown at the bottom left of the screen for 1300 ms. Then a grey box appeared at the bottom right of the screen for 1300 ms and then moved to the left and covered the blue dot array within 1450 ms. After a 650 ms break, a second blue dot array appeared at the upper left for 1300 ms and then moved down and behind the grey box within 2250 ms. Another break of 1300 ms followed, and then a red dot array appeared in the upper right part of the screen for 1300 ms and then moved down next to the grey box within 2250 ms. After another break of 650 ms, a plain grey screen was shown and children had to decide whether the two blue dot arrays behind the grey box or the red dot array contained more dots. After the answer was given orally by the child and protocoled by the examiner, the next trial started. The number of dots within the blue dot arrays ranged from 7 to 34 dots, while the red dot array contained between 18 and 56 dots. In total, there were 20 test trials.

In half of the trials, the two blue arrays contained more dots, and in the other half the red array contained more dots. Dot arrays were created using NASCO (Guillaume et al., 2020) and were controlled for individual size of the dots and mean occupancy in half of the trials. In this case, mean occupancy refers to the average space that each dot takes up within the convex hull (the minimal convex polygon that contains all dots). In the other half of the trials, the dot arrays were controlled for the total area occupied by the dots and their convex hull. The ratios of the sum of the two blue dot arrays to the red dot array were identical to those used in the *non-symbolic comparison* task (3.0, 2.0, 1.5, 1.3 and 1.2). Furthermore, in half of the trials the first blue dot array contained more dots than the second blue dot array. In the other half, the second blue dot array contained more dots than the first blue dot array. Item order was the same for all participants. For instruction, two practice trials were used to explain the task to participants. In these practice trials, the ratio between blue and red dots was 3.0. The examiner gave feedback only for these two trials.

For data analyses, we used the percentage of correct answers across the 20 test trials as a measure for *approximate addition* performance. Internal consistency was α = 0.38 for first grade, α = −0.07 for second grade, α = 0.44 for third grade, and α = 0.71 for fourth grade^1^. Again, some of these reliabilities are low which is common for tasks assessing ANS in children (e.g., Dietrich et al., 2015; Krajcsi et al., 2024) and thus our results concerning approximate addition need to be interpreted cautiously.

#### 2.2.3. Tasks Assessing Symbolic Numerical Skills

For the *symbolic magnitude comparison* task, we used a custom-made tablet application designed in PsychoPy (Peirce et al., 2019) on a 14″ tablet. Pairs of Arabic numbers were shown for 2000 ms on a touchscreen (one at the center of the left half and one at the center of the right half of the touchscreen). Children were asked to touch the half of the touchscreen on which the larger number was displayed. There was no time limit for giving the answer. Similar to Fazio et al. (2014), we used the same ratios for the *symbolic magnitude comparison* task as in the *non-symbolic magnitude comparison* task (3.0, 2.0, 1.5, 1.3, and 1.2). Number pairs were chosen based on the quantities used in the *non-symbolic magnitude comparison* task.

Due to time and resource constraints, we could not use the same number of trials in the *symbolic magnitude comparison* task as in the *non-symbolic magnitude comparison* task. As such, five pairs of single-digit numbers, one for each ratio, were shown first followed by 20 pairs of two-digit numbers, four for each ratio (see Appendix A Table A2). Item order was the same for all participants. In the oral instructions, the pair of 5 and 10 was used as a practice trial to explain the task. No feedback was provided on critical trials.

For data analyses, we used the percentage of correct responses across the 25 critical trials as a measure for *symbolic magnitude comparison* performance. Internal consistency was α = 0.82 in first grade, α = 0.72 in second grade, α = 0.80 in third grade, and α = 0.44 in fourth grade.

For the *number line estimation* task, we also used a custom-made tablet application designed in PsychoPy (Peirce et al., 2019) on a 14″ tablet. The use of a touchscreen allowed for a more intuitive response format than the frequently used paper–pencil format or the digital versions on computers without touchscreens (Gimbert et al., 2019; Sasanguie et al., 2016; Fazio et al., 2014; Lee et al., 2022). In each trial, a black 25.5 cm long line was displayed on a white background. There were three trials with target numbers 7, 2 and 4 on a number line from 0 to 10, and six trials with target numbers 42, 4, 18, 2, 71, and 6 on a number line from 0 to 100 (taken from Siegler & Opfer, 2003). Target numbers were presented above the number line at the center of the touchscreen.

Participants had to tap on the line where they thought the target number was located. There was no feedback, and the presentation order of target numbers was the same for all participants. In the instructions, the number line was explained (e.g., “Down here you see a line. At the beginning of the line is 0. At the end of the line is 10.”) to ensure that the whole line was considered (see Morris et al., 2022). Furthermore, two practice trials were used (5 on the number line from 0 to 10 and 50 on the number line from 0 to 100). For data analyses, we considered the mean percentage of absolute error (PAE) across the nine trials as a measure for *number line estimation* performance: PAE = [|Estimate − Actual Number|/Scale of Estimates] × 100 (Gimbert et al., 2019; Cai et al., 2018; Sasanguie et al., 2016; Siegler & Booth, 2004). Internal consistency for the PAE of all nine items was α = 0.59 in first grade, α = 0.64 in second grade, α = 0.56 in third grade, and α = 0.46 in fourth grade.

#### 2.2.4. Taks Assessing Working Memory

Based on Baddeley’s model (2003), the three WM components central executive (CE), phonological loop (PL) and visuo-spatial sketchpad (VSSP) were assessed using the span tasks from the tablet-based, adaptive application EI-MAG (Oesterlen et al., 2016).

Capacity of the CE was measured using the subtests *digit span backwards*, *word span backwards*, *object span*, and *counting span*. For *digit span backwards*, sequences of numbers were presented via audio which afterwards had to be reproduced by tapping the respective digits on a number pad in reversed order. The *word span backwards* worked similarly with monosyllabic words and a picture matrix. In the *object span* task, sequences of objects were shown and children had to decide for each object whether it was animate or inanimate (secondary task). At the end of each sequence, they had to recall all objects in the order of presentation by tapping pictures in a picture matrix (primary task). In the *counting span* subtest, children had to count target objects in pictures and then put in the corresponding number using a number pad (secondary task). At the end of each sequence of pictures, they had to reproduce these numbers in the order of presentation using another number pad (primary task).

The capacity of the PL was assessed using the *digit span* and the *word span* (*monosyllabic* and *trisyllabic*) tasks reflecting forward versions of the *digit span backwards* and the *word span backwards* tasks.

The capacity of the VSSP was assessed using the subtests *matrix* and *corsi block*. In the *matrix* task, 4 × 4 matrices with several black cells were shown. After that, children were presented with a plain, white matrix and had to reproduce the pattern of black cells seen in the previous matrix by tapping on the respective fields. In the *corsi block* task, randomly placed blocks were shown. Successively, a sequence of single blocks temporarily turned orange. Participants had to recall this sequence in the presented order by tapping the corresponding blocks on the screen.

To ensure task understanding, each subtest started with a practice phase of four sequences. In the case that more than two sequences were not solved correctly during the practice phase, the application showed a notification and subtest-specific oral 1:1 instruction was provided by the examiner. The mean length of the two longest correctly recalled sequences was used as subtest span.

For the components CE, PL and VSSP respectively, the mean of the corresponding subtest spans was calculated if at least two subtests were completed. The mean of the component means was then considered to represent an overall WM score. Internal consistency of the overall WM score was α = 0.75 in first grade, α = 0.73 in second grade, α = 0.65 in third grade, and α = 0.79 in fourth grade. These reliabilities are comparable to the ones reported by Oesterlen et al. (2018).

#### 2.2.5. Control Variables

Several studies reported significant sex differences in mathematics skills, with boys showing higher mathematics skills (e.g., von Davier et al., 2024; Niklas & Schneider, 2012). Furthermore, general cognitive ability is often reported as a predictor of mathematics skills (e.g., W. Schneider & Niklas, 2017; Geary, 2011). Therefore, sex and nonverbal reasoning were considered as control variables in the current study. Nonverbal reasoning was assessed using the adaptive subtest on nonverbal intelligence of BUEGA-II (Esser et al., 2021). The percentage of correct answers across the 35 critical trials was used for data analyses. Internal consistency for the nonverbal intelligence subtest of BUEGA-II was α = 0.86 in first grade, α = 0.87 in second grade, α = 0.81 in third grade, and α = 0.84 in fourth grade and thus comparable to what is reported in the manual (Esser et al., 2021).

### 2.3. Procedure

We used a cross-sectional design in our study. All participants first attended a 90 min group session and then a 35 min one-to-one session, in a quiet room at their respective school. For 480 children (98.77% of the sample) both sessions took place within one month. Due to organizational reasons and illness, for five children from third grade, one child from second grade and one child from fourth grade (1.44% of the sample), the two sessions took place within two months. We compared performance of these children with the remaining sample of the respective grades, applying procedures suggested by Crawford and Garthwaite (2002) and Crawford and Howell (1998). There were no significant differences concerning non-symbolic magnitude comparison, approximate addition, symbolic magnitude comparison, number line estimation, WM, nonverbal reasoning, and mathematics skills between these children and those tested within one month’s time. We therefore included these children in the analyzed sample.

In the group session, children completed the subtests of EI-MAG and DEMAT 1+ in first and second grade, DEMAT 2+ in third grade, and DEMAT 4 in fourth grade, respectively. In the one-to-one session, participants completed the subtest nonverbal intelligence of BUEGA-II, then the non-symbolic magnitude comparison task, the number line estimation task, the symbolic magnitude comparison task, and finally the approximate addition task. Data was collected between February and June.

### 2.4. Statistical Analysis

Data analyses were conducted using R (R Core Team, 2025) in the version 4.5.2 and R Studio (Posit Team, 2025) in the version 2025.9.2.418, as well as the packages dplyr (Wickham et al., 2023) in the version 1.1.4, psych (Revelle, 2025) in the version 2.5.6, car (Fox & Weisberg, 2019) in the version 3.1.3 and broom (Robinson et al., 2025) in the version 1.0.10. Descriptive and correlation analyses will be presented first. Due to time and organizational constraints, the subtests *Calculation Chains* and *Inequations* of the DEMAT 1+ were randomly missing for seven children in first grade, and the subtest *Inequations* of the DEMAT 1+ was randomly missing for twelve children in second grade. These missing values were imputed based on the values of the other subtests and participants in the respective grade level.

Subsequently, we conducted a multi-group path analysis based on grade level using the R package lavaan (Rosseel et al., 2025) in the version 0.6.20 with 5000 bootstrap samples to evaluate whether contributions of non-symbolic numerical skills (i.e., non-symbolic magnitude comparison, approximate addition), symbolic numerical skills (i.e., symbolic magnitude comparison, number line estimation), as well as WM to mathematics skills differ across primary school years.

Overall, the aim of a multi-group path analysis is to test whether one global path model fits the data best or if there are paths in this model that vary across different groups (e.g., see Lefcheck, 2021). To do so, two models are estimated: in one model, parameters are constrained to be the same for the whole sample, whereas in the other model, parameters are free to vary across the different groups within the sample. In the case that the latter model fits the data significantly better, this suggests that there are paths in the model that differ across groups (Lefcheck, 2021).

The tested path model consisted of seven direct paths of the predictor variables to the outcome variable mathematics skills and also considered covariances between all eight variables. Goodness-of-fit indices were evaluated based on the criteria suggested by Hu and Bentler (1999): ≥0.95 for the comparative fit index (CFI), <0.06 for the root mean square error of approximation (RSMEA) and <0.08 for the standardized root mean squared residuals (SRMR). For estimating missing values, we used the full information maximum likelihood (FIML) method. Two children had missing values in the approximate addition task. One child had a missing value in number line estimation and in symbolic magnitude comparison each. Two children had missing values for WM. Three children had missing values in the mathematics tests. All values were missing at random.

For the multi-group path analysis, we conducted sensitivity analyses using G*Power 3.1 (Faul et al., 2009) for each grade level with α = 0.05 and a power of 0.90 (Lakens, 2022). This led to a minimum effect size of $f^{2}$ = 0.13 for first grade, $f^{2}$ = 0.18 for second grade, $f^{2}$ = 0.14 for third grade, and $f^{2}$ = 0.22 for fourth grade to be detected significantly by our design. Consequently, the sensitivity analyses indicate that with our sample of children in primary school and the current study design, we should be able to observe small to moderate effect size associations. To investigate potential differences in the path models between grade levels we conducted a $\chi^{2}$-difference test followed by Bonferroni–Holm-corrected pairwise comparison of the paths.

## 3. Results

### 3.1. Descriptive and Correlation Analyses

Descriptive statistics are displayed in Table 1. Most variables were not normally distributed as indicated by Shapiro–Wilk tests. Based on pairwise Mann–Whitney U-Tests and a Bonferroni–Holm correction for multiple testing, children in higher grade levels typically showed significantly better performance with the following exceptions: First and second graders did not differ significantly on the *approximate addition* task (U = 7194, $n_{1}$ = 149, $n_{2}$ = 110, p = .087, r = −0.10). Second and third graders did not differ significantly for *non-symbolic magnitude comparison* (U = 7977.5, $n_{2}$ = 110, $n_{3}$ = 137, p = .421, r = 0.05) and *approximate addition* (U = 6521, $n_{2}$ = 110, $n_{3}$ = 137, p = .064, r = −0.12). Second and fourth graders did not differ significantly on *non-symbolic magnitude comparison* (U = 4760, $n_{2}$ = 110, $n_{4}$ = 90, p = .635, r = −0.03). Third and fourth graders did not differ significantly regarding *nonverbal reasoning* (U = 5904, $n_{3}$ = 137, $n_{4}$ = 90, p = .590, r = −0.04), *non-symbolic magnitude comparison* (U = 5530.5, $n_{3}$ = 137, $n_{4}$ = 90, p = .183, r = −0.09), *approximate addition* (U = 5407.5, $n_{3}$ = 137, $n_{4}$ = 88, p = .184, r = −0.09) and *symbolic magnitude comparison* (U = 5799, $n_{3}$ = 137, $n_{4}$ = 89, p = .465, r = −0.04).

As grade-specific versions of the DEMAT were used, the comparison of the DEMAT performances across grade levels is not meaningful.

Results of the correlation analyses are summarized in Table 2 and Table 3. In the following, we describe all correlations that differed significantly between grade levels using the procedure suggested by Eid et al. (2017) after applying Bonferroni-Holm correction for multiple testing.

The correlation between sex and *number line estimation* performance was significantly larger in first compared to second, third, and fourth grade. In first, second, and third grade, boys compared to girls showed significantly better *number line estimation* performance. The correlation became weaker the higher the grade level was. In fourth grade, no significant sex differences were found.

Additionally, the correlation between sex and *symbolic magnitude comparison* performance was significantly larger in first than third grade. In first grade, boys were significantly better in *symbolic magnitude comparison*, whereas in third grade no significant correlation with sex was found.

The correlation between *non-symbolic magnitude comparison* performance and *mathematics skills* was also significantly more pronounced in first as compared to third grade. In first grade, children with better *non-symbolic magnitude comparison* performance had significantly better *mathematics skills*. In third grade, this correlation was not significant.

The correlation between *symbolic magnitude comparison* performance and *number line estimation* performance was significantly larger in first grade compared to all other grade levels. Closer inspection of the correlation coefficients indicated that the correlation decreased with increasing grade level. In first and second grade, children with better performance in *number line estimation* showed significantly better performance in *symbolic magnitude comparison*. In third and fourth grade, this correlation was not significant.

The correlation between *symbolic magnitude comparison* performance and *mathematics skills* in first grade was significantly more pronounced than in third and fourth grade. In first, second and third grade, children with higher *symbolic magnitude comparison* performance also showed significantly better *mathematics skills*. The higher the grade level, the weaker this correlation was. In fourth grade, it was not significant.

### 3.2. Multi-Group Path Analysis

**Figure 1:**
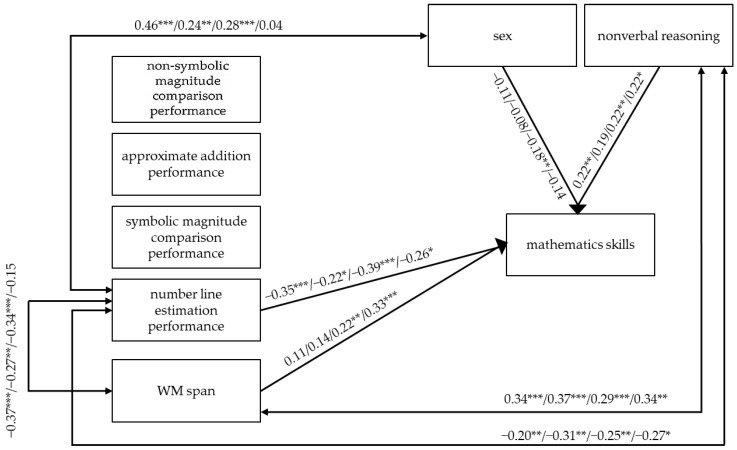
Multi-group path model with performance in *non-symbolic magnitude comparison*, *approximate addition*, *symbolic magnitude comparison*, *number line estimation*, and *WM span* as well as sex and performance in *nonverbal reasoning* as predictors of *mathematics skills* in first, second, third, and fourth grade. *Notes.* Standardized regression coefficients presented for first, second, third, and fourth grade, respectively. Paths that were not significant in any grade level are not displayed for the sake of clarity. Covariances are displayed for significant predictors. Sex was coded 0 for male participants and 1 for female participants. We tested for potential suppression effects by running different versions of the path model (for details please see Appendix A Table A3). Excluding either *nonverbal reasoning* or *number line estimation* from the model led to *WM* becoming a significant predictor also in first and second grade. * p < .05, ** p < .01, *** p < .001.

We conducted a multi-group path analysis (see Figure 1). Variance inflation factors (VIF) for the independent variables performance in *non-symbolic magnitude comparison*, *approximate addition*, *symbolic magnitude comparison*, *number line estimation*, and *WM* capacity as well as sex and performance in *nonverbal reasoning* were between 1.09 and 1.67 across all four grade levels and therefore indicated no issues with multicollinearity in our data set (see, e.g., Salmerón et al., 2018). The goodness-of-fit indices indicated good model fit, $\chi^{2}$(4) = 4.53, p = .339; robust CFI = 0.999, robust RSMEA = 0.033, SRMR = 0.020.

In first grade, performance in *number line estimation* and *nonverbal reasoning* were significant predictors of mathematics skills. Children with better *number line estimation* skills and/or higher skills in *nonverbal reasoning* had better *mathematics skills*. In total, 50.3% of the variance in *mathematics skills* was explained by the path model in first grade.

In second grade, *number line estimation* performance was a significant predictor of *mathematics skills*, explaining 35.6% of the variance in *mathematics skills*. Children with better *number line estimation* skills had better *mathematics skills*.

In third grade, *number line estimation* performance, *WM* span, sex, and *nonverbal reasoning* performance were significant predictors of *mathematics skills* and accounted for 46.3% of the variance in *mathematics skills*. Children with better *number line estimation* skills, higher *WM* capacity, and/or higher skills in *nonverbal reasoning* had better *mathematics skills*. Furthermore, boys had better *mathematics skills* than girls.

In fourth grade, *number line estimation* performance, *WM* span, and *nonverbal reasoning* performance significantly predicted *mathematics skills*, explaining 38.0% of the variance. Children with better *number line estimation* skills, higher *WM* capacity and/or higher skills in *nonverbal reasoning* had better *mathematics skills*.

**Figure 2:**
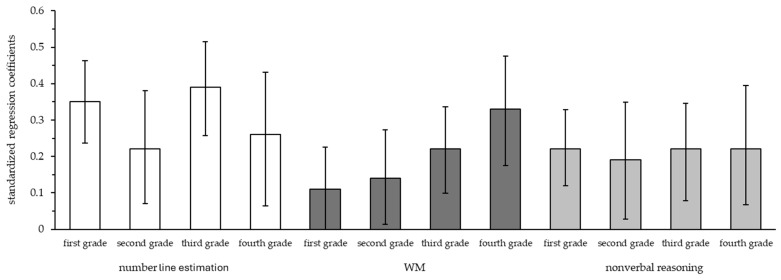
Standardized regression coefficients of the predictors *number line estimation* performance (on the left), *WM* span (in the middle), and *nonverbal reasoning* performance (on the right) throughout primary school. *Notes.* The regression coefficients for *number line estimation* were multiplied by −1 for the sake of comparability. Error bars represent the 90% confidence interval of the standardized regression coefficients.

The standardized regression coefficients of *number line estimation* performance, WM span and *nonverbal reasoning* performance for all four grade levels are displayed in Figure 2.

A $\chi^{2}$-difference test indicated significant differences in path coefficients between grade levels, $\chi^{2}$(45) = 616.00, p < .001. Bonferroni–Holm-corrected pairwise comparison of all paths in all grade levels suggested significant differences concerning the regression weights for *number line estimation* performance predicting *mathematics skills* between first and third grade as well as second and third grade ($\beta_{difference\;between\;grade\;1\;and\;3}$ = 0.05, p = .0099, $\beta_{difference\;between\;grade\;2\;and\;3}$ = 0.18, p = .001). Closer inspection of beta weights indicated that *number line estimation* performance was a significantly stronger predictor of *mathematics skills* in third grade than in first and second grade.

## 4. Discussion

The aim of this study was to jointly evaluate contributions of non-symbolic numerical skills, symbolic numerical skills and WM to mathematics skills and potential developmental changes in these contributions during primary school years. We expected that contributions of non-symbolic numerical skills should decrease with increasing grade level (H1). Contributions of symbolic numerical skills should increase with grade level, reflecting effects of schooling (H2). Overall, we expected stronger contributions of symbolic as compared to non-symbolic numerical skills in primary school children (H3). Furthermore, we expected consistent contributions of WM over the whole period of primary school (H4). In the following, we will address and discuss the contributions of non-symbolic and symbolic numerical skills as well as WM to mathematics skills.

With respect to non-symbolic numerical skills, neither magnitude comparison nor approximate addition were found significant predictors of mathematics skills in any grade level investigated. This adds to the debate about the role of non-symbolic numerical skills and the ANS in the development of mathematics skills (see Chen & Li, 2014 for an overview). Dietrich et al. (2015) suggested that methodological differences, especially regarding the tasks used for examining ANS accuracy, may contribute to heterogenous findings on whether ANS plays a significant role in the development of mathematics skills or not. Taking this into account, we used two separate tasks to assess ANS accuracy, a non-symbolic magnitude comparison task and an approximate addition task. But non-symbolic numerical skills measured by both tasks were not a significant predictor of overall mathematics skills. This partly supports our hypothesis that contributions of non-symbolic numerical skills should decrease with higher grade level given that respective influences have primarily been found before the onset of formal schooling (Cai et al., 2018; Gimbert et al., 2019; but see Halberda et al., 2008).

In contrast, for symbolic numerical skills, we observed that symbolic magnitude comparison was not a significant predictor of mathematics skills in all four grade levels. Interestingly, for children in first grade, there seemingly was a suppression effect with associations of the more complex number line estimation (and of WM) partly accounting for associations of more basic symbolic magnitude comparison. This suppression effect might explain why symbolic magnitude comparison was not a significant predictor of mathematics skills in first grade. In second, third, and fourth grade, there were no comparable suppression effects. Nevertheless, it is important to note that in these grade levels, there seemed to be ceiling effects observed for symbolic magnitude comparison, which suggests that the task was easy to solve for older children in our sample.

However, number line estimation was a significant predictor of mathematics skills in all four grade levels throughout primary school. This is in line with earlier results such as those by Gimbert et al. (2019) who found that number line estimation was a significant predictor of mathematics skills in preschool and third grade (at 5 and 7 years of age). Dietrich et al. (2016) found the same for children in first grade (at 6 years of age) and Siegler and Booth (2004) for children in kindergarten, first and second grade (at 5, 6, and 7 years of age, respectively). Consistently, more accurate number line estimations were predictive of better acquisition of more advanced mathematics skills (see M. Schneider et al., 2018 for a meta-analysis). The substantiated predictive power of number line estimation may result from it being a complex task that builds on and recruits different, more basic mathematics skills (see M. Schneider et al., 2018 for an overview) to, for instance, successfully apply proportion judgement strategies. The latter involves the use of reference points like the start, mid, and end point of the number line to allow better estimation of the position of the target number on the number line. To employ such a strategy, children first need to half the number line to establish the mid point, then decide whether the target number is smaller or larger than the reference point and then estimate the distance on the number line from the reference point to correctly locate the target number (cf. Link et al., 2014). All of these steps rely on basic mathematics skills, thus explaining why number line estimation may be such a strong predictor of mathematics skills in all four grade levels of primary school.

Pairwise multi-group comparison of the predictive power of number line estimation indicated that the contribution of number line estimation to mathematics skills was significantly stronger in third grade compared to first and second grade. This seems to suggest that contributions of number line estimation to mathematics skills are especially important in third grade. Lyons et al. (2014) examined a variety of basic numerical skills and their contributions to mental arithmetic in children from first up to sixth grade cross-sectionally. In contrast to the present study, number line estimation was a significant predictor in first and second grade. In third grade, number line estimation was not a significant predictor. However, in fourth, fifth, and sixth grade number line estimation was again a significant predictor, but less important than in first and second grade. It is important to note, however, that Lyons et al. (2014) had mental arithmetic as an outcome variable, while we focused on a broader range of (more complex) mathematics skills. But, our assumption that number line estimation is especially important for mathematics skills in third grade is in line with the results by Hischa et al. (2025), who found that number line estimation was a significantly more important predictor of mathematics skills in third grade than in preschool (at 9 and 6 years of age, respectively). This may well be explained by the fact that only after starting primary school children regularly deal with symbolic numerical representations (including scaled number lines), which in turn may increase the predictive power of symbolic numerical skills in general and number line estimation in particular (M. Schneider et al., 2018). Number line estimation being most important for mathematics skills in third grade may therefore reflect effects of schooling. For fourth grade the contribution of number line estimation was descriptively, but not significantly smaller than in third grade. This may have to do with the smaller sample size in fourth grade. Accordingly, future research is needed to explore whether this difference is meaningful even though previous studies found number line estimation to significantly predict mathematics skills in fourth grade (e.g., Cai et al., 2018; Zhu et al., 2017).

Finally, WM was a significant predictor of mathematics skills in third and fourth grade. Interestingly, evaluation of potential suppression effects indicated that influences of WM in first and second grade may have been accounted for by nonverbal reasoning. When excluding nonverbal reasoning from the model, WM turned out as a significant predictor for mathematics skills in all four grade levels of primary school. This suppression effect might be explained by the task used to assess, namely the subtest *nonverbal intelligence* of the BUEGA-II (Esser et al., 2021). This subtest is a matrix test requiring participants to recognize colours, shapes, structures, and their relationships as described in the manual. Accordingly, the task puts demands on WM which might explain the observed suppression effects in first and second grade. Consequently, as WM capacity increases with age during childhood, the matrix test might be more sensitive for influences of WM in first and second graders than in third and fourth graders of our sample. Furthermore, when excluding number line estimation from the model, WM again was a significant predictor for mathematics skills in all four grade levels. This finding indicates a suppression effect with associations of number line estimation partly accounting for associations with WM. The number line estimation task was fairly complex and a lot of information had to be processed simultaneously (e.g., Barth & Paladino, 2011; Link et al., 2014; Slusser et al., 2013). The task requires keeping numerical information in mind while estimating its spatial location on a number line. This integration of spatial and numerical information is known to be associated with WM (e.g., Pellizzoni et al., 2025 or see Ünal et al., 2024 for an overview) and might thus explain this suppression effect. The consistent predictive role of WM in all four grade levels in primary school is consistent with earlier findings (see Raghubar et al., 2010 for a review; De Smedt et al., 2009; W. Schneider & Niklas, 2017), which consistently report better WM to predict better mathematics skills. In the long run, greater WM capacity allows processing of more (complex) information and thus may support development of mathematics skills.

### Limitations

When interpreting the current results, the cross-sectional design needs to be noted, which means that we cannot draw strong conclusions on individual development. Additionally, it is important to mention that cross-sectional designs may conflate cohort effects with age effects. However, given the small age range in our sample (each grade is just one year apart), no large cohort effects are to be expected, and there are numerous cross-sectional studies in the field with similar comparisons (e.g., Barth et al., 2006; Gimbert et al., 2019; Halberda & Feigenson, 2008; Li et al., 2018; Slusser et al., 2013). Furthermore, the conducted sensitivity analyses indicated that with the current sample size and design we should be able to observe weak to moderate effects with a power of 0.90. Thus, all observed significant effects should be about this size or larger.

The results of this study have to be interpreted considering at least two limitations, low reliability of some of the tasks used and potential ceiling and floor effects.

Due to time and resource constraints, we only had 40 trials in the non-symbolic magnitude comparison task and 20 trials in the approximate addition task. This may have been one reason for why reliability of these tasks was low for certain grade levels (cf. Dietrich et al., 2015 recommend using more trials to increase reliability). Furthermore, the symbolic comparison task also showed low reliability in fourth grade. Against this background the finding that non-symbolic skills and symbolic comparison were no significant predictors of mathematics skills in the present study needs to be interpreted with caution.

In particular, closer inspection concerning the approximate addition task indicated that the two trial types in this task did not measure the same construct^2^. Consequently, significant contributions of non-symbolic numerical skills to mathematics skills might not have been identified (see Krajcsi et al., 2024). Accordingly, we reran all analyses separately for the two trial types (please see Appendix A Table A4). Importantly, result patterns did not change except for the following: In one multi-group path model, approximate addition was a significant predictor of mathematics skills in fourth grade. In the other model, WM was a significant predictor in second grade, and number line estimation was not a significantly more important predictor in third grade than in first grade.

Furthermore, there were indications for ceiling and floor effects. Non-symbolic magnitude comparison in all four grade levels, approximate addition in first and third grade and symbolic magnitude comparison in second, third, and fourth grade may have been easy to solve for participants in our study, which could possibly explain why performance in these tasks was not a significant predictor of mathematics skills. However, previous studies used similar tasks, which were comparably difficult (see Elliott et al., 2019; Libertus et al., 2013a; Libertus et al., 2013b; Libertus et al., 2011; Van Herwegen et al., 2017; Fazio et al., 2014). For number line estimation, there were indications for floor effects and thus the observed contributions may still be underestimated. In second and third grade there were indications for ceiling effects in the test for mathematics skills. These subsamples were slightly outside the supposed age range for DEMAT 1+ and 2+ (Krajewski et al., 2020; Krajewski et al., 2021). As the DEMATs are the only series of standardized tests for mathematics skills in Germany that are available in a version for each grade level of primary school, we still decided to use them in our study.

## 5. Conclusions

The aim of the current study was to jointly evaluate contributions of non-symbolic and symbolic numerical skills as well as WM to mathematics skills throughout primary school. Results suggested that non-symbolic numerical skills did not significantly contribute to mathematics skills in our sample. In terms of symbolic numerical skills, number line estimation was a significant predictor of mathematics skills in all grade levels, even though this was not the case for symbolic magnitude comparison. Pairwise comparisons of the paths in the multi-group path model revealed that the contribution of number line estimation was more important in third grade compared to first and second grade. Furthermore, WM was a significant predictor of mathematics skills across primary school grades (after considering potential suppression effects for first and second grade). Based on our results, number line estimation seems particularly sensitive to formal instruction and therefore suitable for training (e.g., for the digital context see Ninaus et al., 2017; for the embodied context see Link et al., 2013) and to support further development of mathematics skills.

## Figures and Tables

**Table 1:** Descriptive statistics for sex, performance in *nonverbal reasoning*, *non-symbolic magnitude comparison*, *approximate addition*, *symbolic magnitude comparison*, *number line estimation*, *WM span*, and *mathematics skills* in first, second, third, and fourth grade.

Variables	Grade 1		Grade 2		Grade 3		Grade 4	
	*M*	*SD*	*M*	*SD*	*M*	*SD*	*M*	*SD*
1. Sex	0.54	0.50	0.45	0.50	0.55	0.50	0.56	0.50
2. Nonverbal reasoning performance	44.45	14.43	49.79	15.55	59.12	13.06	59.94	14.87
3. Non-symbolic magnitude comparison performance	90.18	11.30	94.48	4.81	93.87	5.52	95.00	4.00
4. Approximate addition performance	81.28	10.00	83.73	7.21	85.04	9.38	86.99	7.53
5. Symbolic magnitude comparison performance	76.05	17.42	93.42	8.83	96.70	7.37	97.80	3.81
6. Number line estimation performance	14.90	6.16	9.43	4.32	6.29	3.40	4.93	2.16
7. WM span	2.94	0.51	3.27	0.58	3.68	0.57	3.97	0.69
8. Mathematics skills	58.09	21.47	85.27	13.80	74.48	23.81	55.33	18.72

*Notes*. Sex was coded 0 for male participants and 1 for female participants. WM: working memory.

**Table 2:** Correlation coefficients between sex, performance in *nonverbal reasoning*, *non-symbolic magnitude comparison*, *approximate addition*, *symbolic magnitude comparison*, *number line estimation*, *WM span*, and *mathematics skills* in first and second grade.

	1.	2.	3.	4.	5.	6.	7.	8.
1. Sex	—	0.10	−0.02	0.13	−0.19 *	0.23 *	0.13	−0.05
2. Nonverbal reasoning performance	0.08	—	0.30 **	0.09	0.18	−0.27 **	0.33 ***	0.35 ***
3. Non-symbolic magnitude comparison performance	0.01	0.35 ***	—	0.19	0.09	−0.15	0.18	0.23 *
4. Approximate addition performance	−0.05	0.29 ***	0.30 ***	—	0.20 *	−0.03	0.19	0.24 *
5. Symbolic magnitude comparison performance	−0.40 ***	0.18 *	0.31 ***	0.28 ***	—	−0.21 *	0.17	0.30 **
6. Number line estimation performance	0.48 ***	−0.12	−0.28 ***	−0.24 **	−0.56 ***	—	−0.29 **	−0.48 ***
7. WM span	−0.03	0.31 ***	0.34 ***	0.13	0.41 ***	−0.36 ***	—	0.35 ***
8. Mathematics skills	−0.29 ***	0.40 ***	0.42 ***	0.35 ***	0.49 ***	−0.54 ***	0.40 ***	—

*Notes*. Correlation coefficients for first grade are given below the diagonal, correlation coefficients for second grade above the diagonal. Two-tailed bivariate nonparametric correlations (Spearman’s rho). Sex was coded 0 for male participants and 1 for female participants. * p < .05, ** p < .01, *** p < .001.

**Table 3:** Correlation coefficients between sex, performance in *nonverbal reasoning*, *non-symbolic magnitude comparison*, *approximate addition*, *symbolic magnitude comparison*, *number line estimation*, *WM span*, and *mathematics skills* in third and fourth grade.

	1.	2.	3.	4.	5.	6.	7.	8.
1. Sex	—	0.11	−0.05	−0.15	−0.20	−0.06	−0.08	−0.13
2. Nonverbal reasoning performance	0.11	—	0.21 *	0.15	0.04	−0.22 *	0.33 **	0.39 ***
3. Non-symbolic magnitude comparison performance	0.25 **	0.29 ***	—	0.34 **	0.11	−0.13	0.31 **	0.17
4. Approximate addition performance	−0.003	0.03	0.09	—	0.04	−0.06	0.22 *	0.25 *
5. Symbolic magnitude comparison performance	−0.03	0.11	0.16	0.14	—	−0.02	0.17	0.05
6. Number line estimation performance	0.22 **	−0.20 *	−0.03	−0.25 **	−0.16	—	−0.20	−0.32 **
7. WM span	−0.06	0.27 **	0.23 **	0.12	0.20 *	−0.37 ***	—	0.44 ***
8. Mathematics skills	−0.29 ***	0.31 ***	0.12	0.13	0.21 *	−0.52 ***	0.47 ***	—

*Notes*. Correlation coefficients for third grade are given below the diagonal, correlation coefficients for fourth grade above the diagonal. Two-tailed bivariate nonparametric correlations (Spearman’s rho). Sex was coded 0 for male participants and 1 for female participants. * p < .05, ** p < .01, *** p < .001.

## Data Availability

Data and code are provided via OSF: https://doi.org/10.17605/OSF.IO/XJT5M.

## Notes

1 Internal consistency of the *approximate addition* task in second grade was close to zero and negative. Closer inspection indicated that for trials in which the blue dot arrays contained more dots, internal consistency was α = 0.39 in first grade, α = 0.07 in second grade, α = 0.26 in third grade, and α = 0.51 in fourth grade. For trials in which the red dot array contained more dots, internal consistency was α = 0.57, α = 0.41, α = 0.38, and α = 0.68, respectively. In first, third, and fourth grade, performances on these two types of trials did not correlate. In second grade, performance correlated significantly negatively. This indicates that the two trial types may not have measured the same concept in second grade. This was unexpected, as this kind of *approximate addition* task had been used in several other studies (e.g., Barth et al., 2005; Coolen et al., 2022; Gilmore et al., 2010; Iuculano et al., 2008).

2 See Note 1.

## Abbreviations

The following abbreviations are used in this manuscript:

ANS	Approximate number system
CE	Central executive
PAE	Percentage of absolute error
PL	Phonological loop
VSSP	Visuo-spatial sketchpad
WM	Working memory

## Appendix A

**Table A1:** Subtests of the DEMAT tests and their descriptions.

DEMAT Test	Subtest	Description
DEMAT 1+ (Krajewski et al., 2021)	Quantities - Numbers	e.g., splitting a visually presented group of objects into two equal sets
	Number Range	e.g., placing a target number on a scaled number line
	Addition	addition tasks
	Subtraction	subtraction tasks
	Number Decomposition – Number Composition	placing missing numbers in an equation
	Part-Whole Relations	placing missing numbers in an equation of terms
	Calculation Chains	adding several addends
	Inequations	placing the correct (in-)equation sign between a term and a number
	Word Problems	orally presented word problems
DEMAT 2+ (Krajewski et al., 2020)	Number Properties	e.g., marking even numbers
	Comparing Lengths	comparing lengths by placing a suitable (in-)equation sign between them
	Addition	addition tasks
	Subtraction	subtraction tasks
	Duplication	multiplying numbers by 2
	Division	division tasks
	Bisection	dividing numbers by 2
	Calculating with Money	subtraction tasks using amounts of money
	Word Problems	word problems
	Geometry	counting cubes in cube constructions
DEMAT 4 (Gölitz et al., 2006)	Number Lines	choosing the corresponding number to a marked position on an unscaled number line
	Additions	filling out missing places in a written addition problem
	Subtractions	filling out missing places in a written subtraction problem
	Multiplications	multiplication tasks
	Divisions	division tasks
	Comparing Quantities	placing the correct (in-)equation sign between two quantities with units
	Word Problems	word problems
	Spatial Relations	choosing the correct representation of the back view of a given figure
	Reflected Figures	reflecting figures across a given line

**Table A2:** Items of the *symbolic magnitude comparison* task.

	Numbers Presented on the Left	Numbers Presented on the Right
Single-digit number pairs	3	2
	8	4
	6	5
	9	7
	2	6
Two-digit number pairs	29	87
	84	56
	30	15
	62	80
	42	14
	51	34
	69	84
	45	37
	57	74
	49	98
	27	18
	39	51
	96	79
	72	24
	27	54
	53	44
	48	72
	54	18
	69	90
	36	72

**Table A3:** Suppression Analyses.

		Model Excluding NR		Model Excluding NSC		Model Excluding AA		Model Excluding SC		Model Excluding NLE		Model Excluding WM		Model Without Exclusions	
		β	$R^{2}$	β	$R^{2}$	β	$R^{2}$	β	$R^{2}$	β	$R^{2}$	β	$R^{2}$	β	$R^{2}$
first grade			0.45		0.49		0.50		0.50		0.43		0.50		0.50
	sex	−0.08		−0.10		−0.11		−0.15 *		−0.23 **		−0.10		−0.11	
	NR			0.23 ***		0.24 ***		0.22 **		0.22 **		0.25 ***		0.22 **	
	NSC	0.15 *				0.13		0.13		0.16 *		0.11		0.12	
	AA	0.13		0.10				0.10		0.13		0.08		0.09	
	SC	0.13		0.14		0.13				0.20 *		0.16 *		0.12	
	NLE	−0.35 ***		−0.37 ***		−0.36 ***		−0.38 ***				−0.38 ***		−0.35 ***	
	WM	0.18 **		0.10		0.11		0.14 *		0.18 **				0.11	
second grade			0.32		0.35		0.34		0.31		0.32		0.34		0.36
	sex	−0.05		−0.08		−0.07		−0.13		−0.14		−0.05		−0.08	
	NR			0.20 *		0.19		0.23 *		0.22 *		0.23 *		0.19	
	NSC	0.09				0.06		0.05		0.05		0.05		0.04	
	AA	0.13		0.14				0.17		0.12		0.15		0.13	
	SC	0.28		0.25		0.27				0.27		0.26		0.25	
	NLE	−0.25 **		−0.22 *		−0.21 *		−0.24 *				−0.25 **		−0.22 *	
	WM	0.19 *		0.14		0.16 *		0.16		0.19 *				0.14	
third grade			0.41		0.46		0.46		0.46		0.34		0.42		0.46
	sex	−0.16 *		−0.18 **		−0.18 **		−0.18 **		−0.27 ***		−0.19 **		−0.18 **	
	NR			0.23 **		0.22 **		0.22 **		0.30 ***		0.26 **		0.22 **	
	NSC	0.10				0.04		0.05		−0.004		0.08		0.05	
	AA	−0.06		−0.02				−0.03		0.03		−0.03		−0.03	
	SC	0.02		0.02		−0.003				−0.02		0.03		0.01	
	NLE	−0.44 ***		−0.39 ***		−0.39 ***		−0.39 ***				−0.46 ***		−0.39 ***	
	WM	0.26 **		0.22 **		0.22 **		0.22 **		0.33 ***				0.22 **	
fourth grade			0.33		0.38		0.37		0.38		0.32		0.29		0.38
	sex	−0.11		−0.14		−0.16		−0.13		−0.14		−0.16		−0.14	
	NR			0.21 *		0.23 *		0.22 *		0.28 **		0.31 **		0.22 *	
	NSC	−0.02				−0.02		−0.05		−0.05		0.002		−0.05	
	AA	0.13		0.10				0.12		0.10		0.16		0.11	
	SC	−0.06		−0.07		−0.07				−0.04		−0.02		−0.06	
	NLE	−0.32 **		−0.26 *		−0.26 *		−0.26 *				−0.28 *		−0.26 *	
	WM	0.39 ***		0.33 ***		0.35 ***		0.32 ***		0.35 ***				0.33 ***	

*Notes*. Different versions of the multi-group path model, each with one excluded variable, compared to the multi-group path model including all variables. NR: *nonverbal reasoning*; NSC: *non-symbolic comparison*; AA: *approximate addition*; SC: *symbolic comparison*; NLE: *number line estimation*; WM: *working memory*. * *p* < .05, ** *p* < .01, *** *p* < .001.

**Table A4:** Separate multi-group path models for the two trial types of the *approximate addition* task.

		Model Including Only Trials of the AA Task, in Which the Blue Dot Array Contained More Dots			Model Including Only Trials of the AA Task, in Which the Red Dot Array Contained More Dots		
		β		$R^{2}$	β		$R^{2}$
first grade				0.50			0.50
	sex	−	0.11		−	0.11	
	NR		0.23 ***			0.23 **	
	NSC		0.13			0.12	
	AA		0.07			0.04	
	SC		0.13			0.13	
	NLE	−	0.36 ***		−	0.36 ***	
	WM		0.10			0.11	
second grade				0.35			0.35
	sex	−	0.09		−	0.06	
	NR		0.18			0.19	
	NSC		0.05			0.06	
	AA		0.09			0.08	
	SC		0.26			0.27	
	NLE	−	0.22 *		−	0.20 *	
	WM		0.15			0.16 *	
third grade				0.48			0.47
	sex	−	0.18 **		−	0.18 **	
	NR		0.21 **			0.23 **	
	NSC		0.08			0.03	
	AA	−	0.13			0.09	
	SC		0.03		−	0.03	
	NLE	−	0.40 ***		−	0.37 ***	
	WM		0.23 **			0.22 **	
fourth grade				0.43			0.37
	sex	−	0.13		−	0.15	
	NR		0.22 *			0.23 *	
	NSC	−	0.07		−	0.02	
	AA		0.26 **			0.07	
	SC	−	0.07		−	0.08	
	NLE	−	0.26 *		−	0.26 *	
	WM		0.33 ***			0.35 ***	

*Notes*. Comparison of the results of the separate multi-group path models for the two trial types of the *approximate addition* task (trials, in which the blue dot arrays contained more dots vs. trials, in which the red dot arrays contained more dots). NR: *nonverbal reasoning*; NSC: *non-symbolic comparison*; AA: *approximate addition*; SC: *symbolic comparison*; NLE: *number line estimation*; WM: *working memory*. * *p* < .05, ** *p* < .01, *** *p* < .001.

## References

[B1-jintelligence-14-00115] Baddeley A. D. (2003). Working memory: Looking back and looking forward. Nature Reviews Neuroscience.

[B2-jintelligence-14-00115] Bailey D. H., Siegler R. S., Geary D. C. (2014). Early predictors of middle school fraction knowledge. Developmental Science.

[B3-jintelligence-14-00115] Barth H. C., La Mont K., Lipton J., Dehaene S., Kanwisher N., Spelke E. S. (2006). Non-symbolic arithmetic in adults and young children. Cognition.

[B4-jintelligence-14-00115] Barth H. C., La Mont K., Lipton J., Spelke E. S. (2005). Abstract number and arithmetic in preschool children. Proceedings of the National Academy of Sciences of the United States of America.

[B5-jintelligence-14-00115] Barth H. C., Paladino A. M. (2011). The development of numerical estimation: Evidence against a representational shift. Developmental Science.

[B6-jintelligence-14-00115] Braeuning D., Hornung C., Hoffmann D., Lambert K., Ugen S., Fischbach A., Schiltz C., Hübner N., Nagengast B., Moeller K. (2021). Long-term relevance and interrelation of symbolic and non-symbolic abilities in mathematical-numerical development: Evidence from large-scale assessment data. Cognitive Development.

[B7-jintelligence-14-00115] Cai D., Zhang L., Li Y., Wei W., Georgiou G. K. (2018). The role of approximate number system in different mathematics skills across grades. Frontiers in Psychology.

[B8-jintelligence-14-00115] Chen Q., Li J. (2014). Association between individual differences in non-symbolic number acuity and math performance: A meta-analysis. Acta Psychologica.

[B9-jintelligence-14-00115] Coolen I. E. J. I., Riggs K. J., Bugler M., Castronovo J. (2022). The approximate number system and mathematics achievement: It’s complicated. A thorough investigation of different ANS measures and executive functions in mathematics achievement in children. Journal of Cognitive Psychology.

[B10-jintelligence-14-00115] Crawford J. R., Garthwaite P. H. (2002). Investigation of the single case in neuropsychology: Confidence limits on the abnormality of test scores and test score differences. Neuropsychologia.

[B11-jintelligence-14-00115] Crawford J. R., Howell D. C. (1998). Comparing an individual’s test score against norms derived from small samples. The Clinical Neuropsychologist.

[B12-jintelligence-14-00115] De Smedt B. (2022). Individual differences in mathematical cognition: A Bert’s eye view. Current Opinion in Behavioral Sciences.

[B13-jintelligence-14-00115] De Smedt B., Janssen R., Bouwens K., Verschaffel L., Boets B., Ghesquière P. (2009). Working memory and individual differences in mathematics achievement: A longitudinal study from first grade to second grade. Journal of Experimental Child Psychology.

[B14-jintelligence-14-00115] De Smedt B., Noël M.-P., Gilmore C. K., Ansari D. (2013). How do symbolic and non-symbolic numerical magnitude processing skills relate to individual differences in children’s mathematical skills? A review of evidence from brain and behavior. Trends in Neuroscience and Education.

[B15-jintelligence-14-00115] Dietrich J. F., Huber S., Dackermann T., Moeller K., Fischer U. (2016). Place-value understanding in number line estimation predicts future arithmetic performance. British Journal of Developmental Psychology.

[B16-jintelligence-14-00115] Dietrich J. F., Huber S., Nuerk H.-C. (2015). Methodological aspects to be considered when measuring the approximate number system (ANS)—A research review. Frontiers in Psychology.

[B17-jintelligence-14-00115] Eid M., Gollwitzer M., Schmitt M. (2017). Statistik und Forschungsmethoden.

[B18-jintelligence-14-00115] Elliott L., Feigenson L., Halberda J., Libertus M. E. (2019). Bidirectional, longitudinal associations between math ability and approximate number system precision in childhood. Journal of Cognition and Development.

[B19-jintelligence-14-00115] Esser G., Wyschkon A., Ballaschk K. (2021). BUEGA-II: Basisdiagnostik umschriebener entwicklungsstörungen im grundschulalter-version II.

[B20-jintelligence-14-00115] Faul F., Erdfelder E., Buchner A., Lang A.-G. (2009). Statistical power analyses using G*Power 3.1: Tests for correlation and regression analyses. Behavior Research Methods.

[B21-jintelligence-14-00115] Fazio L. K., Bailey D. H., Thompson C. A., Siegler R. S. (2014). Relations of different types of numerical magnitude representations to each other and to mathematics achievement. Journal of Experimental Child Psychology.

[B22-jintelligence-14-00115] Feigenson L., Libertus M. E., Halberda J. (2013). Links between the intuitive sense of number and formal mathematics ability. Child Development Perspectives.

[B23-jintelligence-14-00115] Finke S., Freudenthaler H. H., Landerl K. (2020). Symbolic processing mediates the relation between non-symbolic processing and later arithmetic performance. Frontiers in Psychology.

[B24-jintelligence-14-00115] Fox J., Weisberg S. (2019). An R companion to applied regression.

[B25-jintelligence-14-00115] Friso-van den Bos I., Van der Ven S. H. G., Kroesbergen E. H., Van Luit J. E. H. (2013). Working memory and mathematics in primary school children: A meta-analysis. Educational Research Review.

[B26-jintelligence-14-00115] Geary D. C. (2011). Cognitive predictors of achievement growth in mathematics: A 5-year longitudinal study. Developmental Psychology.

[B27-jintelligence-14-00115] Gilmore C. K., McCarthy S. E., Spelke E. S. (2010). Non-symbolic arithmetic abilities and mathematics achievement in the first year of formal schooling. Cognition.

[B28-jintelligence-14-00115] Gimbert F., Camos V., Gentaz E., Mazens K. (2019). What predicts mathematics achievement? Developmental change in 5- and 7-year-old children. Journal of Experimental Child Psychology.

[B29-jintelligence-14-00115] Gölitz D., Roick T., Hasselhorn M. (2006). Demat 4: Deutscher Mathematiktest für vierte Klassen.

[B30-jintelligence-14-00115] Guillaume M., Schiltz C., Van Rinsveld A. (2020). NASCO: A new method and program to generate dot arrays for non-symbolic number comparison tasks. Journal of Numerical Cognition.

[B31-jintelligence-14-00115] Halberda J., Feigenson L. (2008). Developmental change in the acuity of the “number sense”: The approximate number system in 3-, 4-, 5-, and 6-year-olds and adults. Developmental Psychology.

[B32-jintelligence-14-00115] Halberda J., Ly R., Wilmer J. B., Naiman D. Q., Germine L. (2012). Number sense across the lifespan as revealed by a massive internet-based sample. Proceedings of the National Academy of Sciences of the United States of America.

[B33-jintelligence-14-00115] Halberda J., Mazzocco M. M. M., Feigenson L. (2008). Individual differences in non-verbal number acuity correlate with maths achievement. Nature.

[B34-jintelligence-14-00115] Halberda J., Odic D., Geary D. C., Berch D. B., Mann Koepke K. (2015). The precision and internal confidence of our approximate number thoughts. Evolutionary origins and early development of number processing.

[B35-jintelligence-14-00115] Hischa V., Moeller K., Seitz-Stein K., Niklas F. (2025). Differential contributions of approximate number system, number line estimation, and working memory to mathematical skills in preschool and primary school. Psychological Research.

[B36-jintelligence-14-00115] Hornung C., Schiltz C., Brunner M., Martin R. (2014). Predicting first-grade mathematics achievement: The contributions of domain-general cognitive abilities, nonverbal number sense, and early number competence. Frontiers in Psychology.

[B37-jintelligence-14-00115] Hu L., Bentler P. M. (1999). Cutoff criteria for fit indexes in covariance structure analysis: Conventional criteria versus new alternatives. Structural Equation Modeling: A Multidisciplinary Journal.

[B38-jintelligence-14-00115] Iuculano T., Tang J., Hall C. W. B., Butterworth B. (2008). Core information processing deficits in developmental dyscalculia and low numeracy. Developmental Science.

[B39-jintelligence-14-00115] Krajcsi A., Chesney D., Cipora K., Coolen I. E. J. I., Gilmore C. K., Inglis M., Libertus M. E., Nuerk H.-C., Simms V., Reynvoet B. (2024). Measuring the acuity of the approximate number system in young children. Developmental Review.

[B40-jintelligence-14-00115] Krajewski K., Dix S., Schneider W. (2020). Demat 2+: Deutscher Mathematiktest für zweite Klassen.

[B41-jintelligence-14-00115] Krajewski K., Küspert P., Schneider W. (2021). Demat 1+: Deutscher Mathematiktest für erste Klassen.

[B42-jintelligence-14-00115] Lakens D. (2022). Sample size justification. Collabra: Psychology.

[B43-jintelligence-14-00115] Lee S. H., Kim D., Opfer J. E., Pitt M. A., Myung J. I. (2022). A number-line task with a Bayesian active learning algorithm provides insights into the development of non-symbolic number estimation. Psychonomic Bulletin & Review.

[B44-jintelligence-14-00115] Lefcheck J. S. (2021). An introduction to structural equation modeling.

[B45-jintelligence-14-00115] Li Y., Zhang M., Chen Y., Deng Z., Zhu X., Yan S. (2018). Children’s non-symbolic and symbolic numerical representations and their associations with mathematical ability. Frontiers in Psychology.

[B46-jintelligence-14-00115] Libertus M. E., Feigenson L., Halberda J. (2011). Preschool acuity of the approximate number system correlates with school math ability. Developmental Science.

[B47-jintelligence-14-00115] Libertus M. E., Feigenson L., Halberda J. (2013a). Is approximate number precision a stable predictor of math ability?. Learning and Individual Differences.

[B48-jintelligence-14-00115] Libertus M. E., Feigenson L., Halberda J. (2013b). Numerical approximation abilities correlate with and predict informal but not formal mathematics abilities. Journal of Experimental Child Psychology.

[B49-jintelligence-14-00115] Link T., Moeller K., Huber S., Fischer U., Nuerk H.-C. (2013). Walk the number line—An embodied training of numerical concepts. Trends in Neuroscience and Education.

[B50-jintelligence-14-00115] Link T., Nuerk H.-C., Moeller K. (2014). On the relation between the mental number line and arithmetic competencies. Quarterly Journal of Experimental Psychology.

[B51-jintelligence-14-00115] Lyons I. M., Price G. R., Vaessen A., Blomert L., Ansari D. (2014). Numerical predictors of arithmetic success in grades 1–6. Developmental Science.

[B52-jintelligence-14-00115] Malone S. A., Burgoyne K., Hulme C. (2020). Number knowledge and the approximate number system are two critical foundations for early arithmetic development. Journal of Educational Psychology.

[B53-jintelligence-14-00115] Malone S. A., Pritchard V. E., Heron-Delaney M., Burgoyne K., Lervåg A., Hulme C. (2019). The relationship between numerosity discrimination and arithmetic skill reflects the approximate number system and cannot be explained by inhibitory control. Journal of Experimental Child Psychology.

[B54-jintelligence-14-00115] Martin R. B., Cirino P. T., Sharp C., Barnes M. A. (2014). Number and counting skills in kindergarten as predictors of grade 1 mathematical skills. Learning and Individual Differences.

[B55-jintelligence-14-00115] Mazzocco M. M. M., Feigenson L., Halberda J. (2011). Preschoolers’ precision of the approximate number system predicts later school mathematics performance. PLoS ONE.

[B56-jintelligence-14-00115] Meyer M. L., Salimpoor V. N., Wu S. S., Geary D. C., Menon V. (2010). Differential contribution of specific working memory components to mathematics achievement in 2nd and 3rd graders. Learning and Individual Differences.

[B57-jintelligence-14-00115] Morris B. J., Todaro R., Arner T., Roche J. M. (2022). How does the accuracy of children’s number representations influence the accuracy of their numerical predictions?. Frontiers in Psychology.

[B58-jintelligence-14-00115] Niklas F., Schneider W. (2012). Die Anfänge geschlechtsspezifischer Leistungsunterschiede in mathematischen und schriftsprachlichen Kompetenzen. Zeitschrift für Entwicklungspsychologie und Pädagogische Psychologie.

[B59-jintelligence-14-00115] Ninaus M., Kiili K., McMullen J., Moeller K. (2017). Assessing fraction knowledge by a digital game. Computers in Human Behavior.

[B60-jintelligence-14-00115] Obersteiner A., Reiss K., Ufer S. (2013). How training on exact or approximate mental representations of number can enhance first-grade students’ basic number processing and arithmetic skills. Learning and Instruction.

[B61-jintelligence-14-00115] Oesterlen E., Eichner M., Gade M., Seitz-Stein K. (2018). Tablet-based working memory assessment in children and adolescents. Zeitschrift für Entwicklungspsychologie und Pädagogische Psychologie.

[B62-jintelligence-14-00115] Oesterlen E., Gade M., Seitz-Stein K. (2016). EI-MAG: Eichstätter Messung des Arbeitsgedächtnisses [Eichstätt Working Memory Assessment: Application]: Unpublished research instrument.

[B63-jintelligence-14-00115] Passolunghi M. C., Lanfranchi S. (2012). Domain-specific and domain-general precursors of mathematical achievement: A longitudinal study from kindergarten to first grade. The British Journal of Educational Psychology.

[B64-jintelligence-14-00115] Passolunghi M. C., Vercelloni B., Schadee H. (2007). The precursors of mathematics learning: Working memory, phonological ability and numerical competence. Cognitive Development.

[B65-jintelligence-14-00115] Peirce J., Gray J. R., Simpson S., MacAskill M., Höchenberger R., Sogo H., Kastman E., Lindeløv J. K. (2019). PsychoPy2: Experiments in behavior made easy. Behavior Research Methods.

[B66-jintelligence-14-00115] Pellizzoni S., Granello F., Cuder A., Doz E., Passolunghi M. C. (2025). Longitudinal relationships between visuospatial working memory, verbal counting and number line knowledge in preschoolers. Psychological Research.

[B67-jintelligence-14-00115] Peng P., Namkung J., Barnes M. A., Sun C. (2016). A meta-analysis of mathematics and working memory: Moderating effects of working memory domain, type of mathematics skill, and sample characteristics. Journal of Educational Psychology.

[B68-jintelligence-14-00115] Posit Team (2025). RStudio: Integrated development environment for R.

[B69-jintelligence-14-00115] Raghubar K. P., Barnes M. A., Hecht S. A. (2010). Working memory and mathematics: A review of developmental, individual difference, and cognitive approaches. Learning and Individual Differences.

[B70-jintelligence-14-00115] R Core Team (2025). R: A language and environment for statistical computing.

[B71-jintelligence-14-00115] Revelle W. (2025). psych: Procedures for psychological, psychometric, and personality research.

[B72-jintelligence-14-00115] Robinson D., Hayes A., Couch S. (2025). broom: Convert statistical objects into tidy tibbles.

[B73-jintelligence-14-00115] Rosseel Y., Jorgensen T. D., De Wilde L. (2025). lavaan: Latent variable analysis.

[B74-jintelligence-14-00115] Salmerón R., García C. B., García J. (2018). Variance inflation factor and condition number in multiple linear regression. Journal of Statistical Computation and Simulation.

[B75-jintelligence-14-00115] Sasanguie D., Verschaffel L., Reynvoet B., Luwel K. (2016). The development of symbolic and non-symbolic number line estimations: Three developmental accounts contrasted within cross-sectional and longitudinal data. Psychologica Belgica.

[B76-jintelligence-14-00115] Schneider M., Beeres K., Coban L., Merz S., Schmidt S. S., Stricker J., De Smedt B. (2017). Associations of non-symbolic and symbolic numerical magnitude processing with mathematical competence: A meta-analysis. Developmental Science.

[B77-jintelligence-14-00115] Schneider M., Merz S., Stricker J., De Smedt B., Torbeyns J., Verschaffel L., Luwel K. (2018). Associations of number line estimation with mathematical competence: A meta-analysis. Child Development.

[B78-jintelligence-14-00115] Schneider W., Niklas F. (2017). Intelligence and verbal short-term memory/working memory: Their interrelationships from childhood to young adulthood and their impact on academic achievement. Journal of Intelligence.

[B79-jintelligence-14-00115] Siegler R. S., Booth J. L. (2004). Development of numerical estimation in young children. Child Development.

[B80-jintelligence-14-00115] Siegler R. S., Braithwaite D. W. (2017). Numerical development. Annual Review of Psychology.

[B81-jintelligence-14-00115] Siegler R. S., Opfer J. E. (2003). The development of numerical estimation: Evidence for multiple representations of numerical quantity. Psychological Science.

[B82-jintelligence-14-00115] Slusser E. B., Santiago R. T., Barth H. C. (2013). Developmental change in numerical estimation. Journal of Experimental Psychology: General.

[B83-jintelligence-14-00115] Starr A., Brannon E. M., Geary D. C., Berch D., Mann Koepke K. (2015). Evolutionary and developmental continuities in numerical cognition. Evolutionary origins and early development of number processing.

[B84-jintelligence-14-00115] Toll S. W. M., Van Viersen S., Kroesbergen E. H., Van Luit J. E. H. (2015). The development of (non-)symbolic comparison skills throughout kindergarten and their relations with basic mathematical skills. Learning and Individual Differences.

[B85-jintelligence-14-00115] Ünal Z. E., Terzi Z., Yalvaç B., Geary D. C. (2024). The relation between number line performance and mathematics outcomes: Two meta-analyses. Developmental Science.

[B86-jintelligence-14-00115] Van Herwegen J., Costa H. M., Passolunghi M. C. (2017). Improving approximate number sense abilities in preschoolers: PLUS games. School Psychology Quarterly.

[B87-jintelligence-14-00115] von Aster M. G., Shalev R. S. (2007). Number development and developmental dyscalculia. Developmental Medicine & Child Neurology.

[B88-jintelligence-14-00115] von Davier M., Kennedy A., Reynolds K., Fishbein B., Khorramdel L., Aldrich C., Bookbinder A., Bezirhan U., Yin L. (2024). TIMSS 2023 international results in mathematics and science.

[B89-jintelligence-14-00115] Vukovic R. K., Fuchs L. S., Geary D. C., Jordan N. C., Gersten R., Siegler R. S. (2014). Sources of individual differences in children’s understanding of fractions. Child Development.

[B90-jintelligence-14-00115] Wickham H., François R., Henry L., Müller K., Vaughan D. (2023). dplyr: A grammar of data manipulation.

[B91-jintelligence-14-00115] Zheng X., Swanson H. L., Marcoulides G. A. (2011). Working memory components as predictors of children’s mathematical word problem solving. Journal of Experimental Child Psychology.

[B92-jintelligence-14-00115] Zhu M., Cai D., Leung A. W. S. (2017). Number line estimation predicts mathematical skills: Difference in grades 2 and 4. Frontiers in Psychology.

